# Reliability and Acute Changes in the Load–Velocity Profile During Countermovement Jump Exercise Following Different Velocity‐Based Resistance Training Protocols in Recreational Runners

**DOI:** 10.1002/ejsc.12309

**Published:** 2025-04-29

**Authors:** Alejandro Pérez‐Castilla, Santiago A. Ruiz‐Alias, Rodrigo Ramirez‐Campillo, Felipe García‐Pinillos, Aitor Marcos‐Blanco

**Affiliations:** ^1^ Department of Education Faculty of Education Sciences University of Almería Almería Spain; ^2^ SPORT Research Group (CTS‐1024) CIBIS (Centro de Investigación para el Bienestar y la Inclusión Social) Research Center University of Almería Almería Spain; ^3^ Department of Physical Education and Sport Faculty of Sport Sciences University of Granada Granada Spain; ^4^ Sport Sciences and Human Performance Laboratories Instituto de Alta Investigación, Universidad de Tarapacá Arica Chile; ^5^ Department of Physical Education Sports and Recreation Universidad de La Frontera Temuco Chile

**Keywords:** force‐velocity relationship, human physical conditioning, muscle function, resistance training

## Abstract

This study aimed (i) to explore the reliability of the load–velocity relationship variables (load‐axis intercept [*L*_0_], velocity‐axis intercept [*v*_0_], and the area under the load–velocity relationship line [*A*_*line*_]) obtained during the countermovement jump exercise in successive sessions and (ii) to examine the feasibility of the load–velocity relationship variables to detect acute changes in the lower‐body maximal mechanical capacities following different velocity‐based training (VBT) protocols. Twenty‐one recreational runners completed four randomized VBT protocols (three back squat sets with three minutes of rest) on separate occasions: (i) VBT with 60% of the one‐repetition maximum (1RM) and 10% velocity loss (VBT_60–10_); (ii) VBT with 60% 1RM and 30% velocity loss (VBT_60–30_); (iii) VBT with 80% 1RM and 10% velocity loss (VBT_80–10_); and (iv) VBT with 80% 1RM and 30% velocity loss (VBT_80–30_). The load–velocity relationship was determined before and after each VBT protocol using the two‐point method in the countermovement jump with a 0.5 kg load and another matching a mean velocity of 0.55 m·s^−1^. All load–velocity relationship variables had an acceptable reliability (CV ≤ 5.61% and ICC ≥ 0.83, except for *v*_0_ between VBT_60–30_ and VBT_80–10_). Both *v*_0_ and *A*_*line*_ were reduced after VBT_60–30_ and VBT_80–30_ (*p* ≤ 0.044 and ES ≥ −0.47) but not after VBT_60–10_ and VBT_80–10_ (*p* ≥ 0.066 and ES ≤ −0.37). The post–pre differences were not significantly associated between VBT protocols for any load–velocity relationship variable (*r* ≤ 0.327 and *p* ≥ 0.148). Although the load–velocity relationship is reliable and sensitive to high‐repetition VBT protocols, its use to detect acute changes in the lower‐body maximal mechanical capacities is characterized by a high variability in individual responses.

**Summary**

- The study confirmed that load–velocity relationship variables (*L*_0_, *v*_0_, and *A*_*line*_) obtained during countermovement jumps exhibit high reliability across sessions. This validates their use as reliable metrics for assessing maximal mechanical capacities in the lower body, particularly in field settings​.
- High‐repetition velocity‐based resistance training (VBT) protocols, particularly those with 30% velocity loss, led to significant reductions in *v*_0_ and *A*_*line*_, indicating a marked decrease in lower‐body neuromuscular capacities. This suggests that these protocols induce greater fatigue than those with a 10% velocity loss, regardless of load magnitude.
- The load–velocity relationship obtained during countermovement jump exercise the can be a useful tool for monitoring fatigue and neuromuscular performance in recreational runners. However, given the high variability in individual responses to different VBT protocols, coaches should interpret changes in performance on a case‐by‐case basis to optimize training outcomes.

## 1 Introduction

Resistance training can improve running performance (Beattie et al. 2017; Blagrove et al. 2018; Jung 2003). However, running performance can be compromised when resistance training is performed before endurance running training (Doma et al. 2017; Doma et al. 2019). This phenomenon has been referred to as *“resistance training‐induced suboptimization on endurance performance”* (RT‐SEP) (Doma et al. 2019). Therefore, it is of paramount importance to manage neuromuscular fatigue from previous resistance training sessions to optimize training stimuli from subsequent running sessions (Doma et al. 2017). A recent systematic review with meta‐analysis conducted with runners (de Carvalho e Silva et al. 2022) reported acute decrease in peak torque of the knee extensor after strength training sessions, although the reduction was low in the countermovement jump test. However, traditional tests (e.g., vertical jumps) do not allow to discern among the maximal mechanical limits of the neuromuscular system to produce force (i.e., force at zero velocity [*F*_*0*_]), velocity (i.e., velocity at zero force [*v*_0_]), and power (i.e., maximum power [*P*_max_]) (Jaric 2016).

Alternatively, the force–velocity (F‐V) relationship can assess the effects of fatigue on the distinctive maximal neuromuscular capacities (García‐Ramos et al. 2018; Li et al. 2024). García‐Ramos et al. (2018) reported a reduction in *P*_max_ due to a deterioration in *v*_0_ after a bench press protocol leading to failure with the 60% of the one‐repetition maximum (1RM) and in *F*_*0*_ after a bench press protocol not leading to failure with the 80% 1RM. Li et al. (2024) also observed a decrease in *P*_max_ due to the muscle inability to develop *v*_0_ or *F*_*0*_ following a heavy‐load traditional (five repetitions at 80% 1RM) or light‐load ballistic (five repetitions at 30% 1RM) squat protocol, respectively. However, the F–V profile is not free of limitations. For example, in resistance exercises performed against gravity (e.g., bench press throw and squat jump), the large extrapolation of the first experimental point to *v*_0_ can compromise the reliability and precision of the F–V relationship parameters (i.e., *v*_0_ and *P*_max_) (Pérez‐Castilla et al. 2021).

To overcome this limitation, some authors have recently determined the load–velocity (L–V) relationship in multiple resistance training exercises (Miras‐Moreno et al. 2023; Pérez‐Castilla et al. 2021; Pérez‐Castilla et al. 2022; Pérez‐Castilla, Ramirez‐Campillo, et al. 2023). This novel procedure only requires the application of a simple regression model to the velocity data recorded under two or more loading conditions to determine the load‐axis intercept (i.e., load at zero velocity [*L*_0_]), velocity‐axis intercept (i.e., *v*_0_), and the area under the L–V relationship line (i.e., *A*_*line*_ = *L*_0_ × *v*_0_/2) (Pérez‐Castilla et al. 2021). Previous research has confirmed the high reliability, sensibility, and concurrent validity of the *L*_0_, *v*_0_, and *A*_*line*_ with respect to traditional tests commonly used to evaluate maximal muscles' capacities to produce force, velocity, and power (Pérez‐Castilla et al. 2021; Pérez‐Castilla et al. 2022; Quidel‐Catrilelbún et al. 2024). Furthermore, from a practical point of view, the L–V relationship variables can be obtained more quickly from the *“two‐point method”* (i.e., only two loads are used to model the L–V relationship) (Miras‐Moreno et al. 2023; Pérez‐Castilla, Ramirez‐Campillo, et al. 2023). Applying this simple testing procedure in the prone bench pull exercise, a recent study (Quidel‐Catrilelbún et al. 2024) has found a significant reduction in *L*_0_ and *A*_*line*_ after different velocity‐based resistance training (VBT) protocols (60% 1RM and 80% 1RM combined with 10% and 30% velocity loss in the set) followed by a 2000 m rowing ergometer time trial in competitive rowers. Therefore, it would be of especial interest for practitioners and coaches to confirm these findings using lower extremity resistance training exercises in other cyclical sports, such as running. It would also be relevant to confirm the between‐session reliability of the two‐point method obtained under field conditions since only one study (Miras‐Moreno et al. 2023) has been conducted to date (in the Smith machine prone bench pull exercise).

Another relevant issue to explore related to the L–V profile is its potential interaction with participant's sex. Several physical capacities often differ between males and females (Besson et al. 2022; Nuzzo 2023). For example, males are more susceptible to an acute loss of force production than females, but these sex‐related differences were diminished after an 8‐week VBT program with 20% or 40% velocity loss (Walker et al. 2022). Furthermore, although males and females achieved similar strength and power performance gains following VBT, females who training with 40% velocity loss tended to benefit more for upper‐body strength and power gains than those who training with 20% velocity loss (Rissanen et al. 2022). These findings suggest that females have greater muscle fatigability than males. Therefore, changes in the L–V profile after different VBT protocols may depend on participant's sex.

Therefore, the objective of the present study was twofold: (i) to explore the reliability of the L–V relationship variables (*L*_0_, *v*_0_, and *A*_*line*_) obtained through the two‐point method applied in field conditions during the countermovement jump exercise in successive sessions and (ii) to examine the feasibility of the L–V relationship variables to detect acute changes in the lower‐body maximal mechanical capacities following four different back squat VBT protocols, in terms of load magnitude (60% 1RM vs. 80% 1RM) and velocity loss in the set (10% vs. 30%), in male and female recreational runners. We hypothesized that (i) the three L–V relationship variables (*L*_0_, *v*_0_, and *A*_*line*_) would have an acceptable between‐day reliability (Miras‐Moreno et al. 2023; Pérez‐Castilla et al. 2022), (ii) the decrease in *A*_*line*_ would be more predominant for high‐repetition VBT protocols through a selective reduction in *L*_0_ (Quidel‐Catrilelbún et al. 2024), and (iii) the changes in the L–V profile would be more pronounced in males than in females (Walker et al. 2022).

## 2 Methods

### 2.1 Experimental Design

A randomized controlled crossover design was used to explore the reliability and feasibility of the L–V relationship variables obtained during the countermovement jump exercise to monitor acute changes in the lower‐body maximal mechanical capacities following different back squat VBT protocols in male and female recreational runners. Subjects completed four different VBT protocols separated by 48–72 h: (i) VBT protocol with 60% 1RM and a velocity loss in the set of 10% (VBT_60–10_), (ii) VBT protocol with 60% 1RM and a velocity loss in the set of 30% (VBT_60–30_), (iii) VBT protocol with 80% 1RM and a velocity loss in the set of 10% (VBT_80–10_), and (iv) VBT protocol with 80% 1RM and a velocity loss in the set of 30% (VBT_80–30_). The block randomization method was used to determine the VBT protocol order for each subject. All sessions were performed at the same time of the day (± 2 h) to minimize the potential influence of diurnal variations on strength performance.

### 2.2 Subjects

Twenty‐one recreational runners, 11 males (age = 28.4 ± 6.4 years [range: 19–38]; body mass = 78.9 ± 11.2 kg; body height = 176.4 ± 6.0 cm; and half back squat 1RM relative to body mass = 1.8 ± 0.4) and 10 females (age = 23.5 ± 2.1 years [range: 21–28]; body mass = 55.2 ± 6.9 kg; body height = 161.2 ± 7.8 cm; and half back squat 1RM relative to body mass = 1.5 ± 0.3), volunteered to participate in this study. Selection criteria were as follows: (i) willing to abstain from any strenuous exercise during the study, (ii) having at least 1 year of resistance training experience, (iii) being familiar with the back squat as part of their usual training, and (iv) being free from musculoskeletal limitations for the last 3 months. Subjects were informed about the purpose and study procedures before signing a written informed consent form. The study protocol adhered to the tenets of the most recent version of the Declaration of Helsinki and was approved by the Institutional Review Board.

### 2.3 Procedures

Body mass and height were measured using a contact electrode foot‐to‐foot body fat analyzer system (TBF‐300A; Tanita Corp of America Inc., Arlington Heights, IL) and a wall‐mounted stadiometer (Seca 202; Seca Ltd., Hamburg, Germany). Each VBT protocol began with the same warm‐up that consisted of jogging, dynamic stretching, and joint mobility exercises, followed by two sets of 10 air squats and five submaximal countermovement jumps. Then, the subjects completed a set of six, four, and two repetitions at 40%, 60%, and 80% of self‐reported back squat 1RM, respectively. The absolute load (kg) corresponding to each relative load was maintained across all sessions. The repetition with a higher mean velocity in each warm‐up set was used to estimate the load equivalent to a mean velocity of 0.55 m·s^−1^ (load_0.55_) in the individualized L–V relationship (Pérez‐Castilla, Ramirez‐Campillo, et al. 2023). A validated linear velocity transducer (T‐Force system; Ergotech, Murcia, Spain) was used to automatically calculate the mean velocity and provide auditory mean velocity feedback after each repetition (Pérez‐Castilla et al. 2019).

After 3 minutes of passive rest (i.e., sitting and slow walking), the L–V relationship was determined using the two‐point method before and after each VBT protocol. Following previous recommendations (Pérez‐Castilla, Ramirez‐Campillo, et al. 2023), three countermovement jumps with a wooden barbell (< 0.5 kg) and two back squats against the load_0.55_ (89.3 ± 29.6 kg [43–170 kg]) were performed separately by three minutes of rest. An additional repetition was performed when the mean velocity differed > 10% between repetitions, and the most extreme value was discarded from the analysis (Baena‐Raya et al. 2024). Specifically, the average score of the mean velocity values collected under the two different loading conditions was used to determine the L–V relationship variables (*L*_0_, *v*_0_, and *A*_*line*_) by applying a least‐square linear regression model (see for (Pérez‐Castilla et al. 2021) for further details).

The VBT protocols were configured using two different relative loads (60% vs. 80% 1RM) and two different velocity loss thresholds in the set (10% vs. 30%). The relative load was determined in each session from the individualized L–V relationship using the specific warm‐up sets and a minimal velocity threshold of 0.33 m·s^−1^ (Pérez‐Castilla et al. 2020). The sets were terminated when the subjects were unable to complete two consecutive repetitions above the velocity loss limit or with the full range of motion (see below for further details). The fastest repetition from the first set was used to define the target velocity loss limit (Quidel‐Catrilelbún et al. 2024). The same exercise (back squat), number of sets (three), and interset rest (3 minutes) were used in all VBT protocols.

Both exercises involved subjects standing with the knees and hips fully extended, feet approximately shoulder width apart, and the barbell held across the top of the shoulders and upper back. From this position, they were required to jump as high as possible after performing a fast countermovement to a self‐selected depth (countermovement jump) or to return to the starting position as fast as possible after performing a continuous downward movement until their buttocks were contacted with a wooden box (back squat) (Morenas‐Aguilar et al. 2023). The height of the wooden box was individually set at 90° of knee flexion with a manual goniometer (Goniómetro Rulong, Fisaude, Spain).

## 3 Statistical Analyses

Descriptive data are presented as mean ± standard deviation (SD). The normality of the data was confirmed using the Shapiro–Wilk test (*p* > 0.05). The between‐day reliability was assessed using the coefficient of variation (CV = standard error of measurement [SEM]/subjects' mean score × 100) and the intraclass correlation coefficient (ICC; Model 3.1) with their corresponding 95% confidence intervals (95% CIs). An acceptable reliability was determined as a CV < 10% and ICC > 0.80 (Lindberg et al. 2021). Additionally, the smallest detectable differences was calculated for each L–V relationship variable as 1.96 × √2 × SEM. A mixed model analysis of variance (ANOVA) was applied to the VBT (load, fastest mean velocity of the first set [MV_fastest_], and total number of repetitions) and L–V relationship (*L*_0_, *v*_0_, and *A*_*line*_) variables, with the protocol (VBT_60–10_ vs. VBT_60–30_ vs. VBT_80–10_ vs. VBT_80–30_) as a within‐subject factor and sex (male vs. female) as a between‐subject factor. A one‐way repeated‐measures ANOVA was also applied to the L–V relationship variables obtained at the beginning of each session and the post–pre differences obtained from each L–V relationship variable. The Greenhouse–Geisser correction was used when Mauchly's sphericity test was violated and pairwise comparisons were identified using Bonferroni *post hoc* corrections. The magnitude of the differences was quantified using the standardized mean differences (Cohen's *d* effect size [ES]) calculated as the raw mean difference divided by the pooled SD of the compared conditions. The ES for changes between VBT protocols for each L–V relationship variable was calculated as mean post–pre differences of one condition—mean post–pre differences of another condition/pooled SD. The following scale was used to interpret the magnitude of the ES: *trivial* (< 0.20), *small* (0.20–0.59), *moderate* (0.60–1.19), *large* (1.20–2.00), and *extremely large* (> 2.00) (Hopkins et al. 2009). Pearson's product‐moment correlation coefficient (*r*) was used to quantify the association for the post–pre differences of each L–V relationship variable between VBT protocols. All reliability assessments were performed using a custom Excel spreadsheet (Hopkins 2015). Other statistical analyses were performed using the SPSS software package (IBM SPSS version 25.0, Chicago, IL, USA). Statistical significance was established at an alpha level of 0.05.

## 4 Results

### 4.1 Characteristics of VBT Protocols

A main effect of protocol was reported for load, MV_fastest_, and total number of repetitions (F_(3,57)_ ≥ 54.26 and *p* < 0.001) as well as of sex for load (F_(1,19)_ ≥ 27.93 and *p* < 0.001). The main effects showed that (i) the load was higher for VBT_80–10_ and VBT_80–30_ than for VBT_60–10_ and VBT_60–30_ (*p* < 0.001 and ES ≥ 1.50), (ii) the load was higher for males than for females (*p* < 0.001 and ES = 1.92), (iii) the MV_fastest_ was higher for VBT_60–10_ and VBT_60–30_ than for VBT_80–10_ and VBT_80–30_ (*p* < 0.001 and ES ≥ 1.42), and (iv) the total number of repetitions was higher for VBT_60–30_, followed by VBT_60–10_, VBT_80–30_, and VBT_80–10_ (*p* ≤ 0.002 and ES ≥ 0.77), except between VBT_60–10_ and VBT_80–30_ (*p* = 1.000 and ES = 0.22). The protocol × sex interaction also reached statistical significance for load (F_(3,57)_ = 8.41 and *p* < 0.001) and MV_fastest_ (F_(3,57)_ = 7.23 and *p* < 0.001). Specifically, the load and MV_fastest_ tended to be slightly greater for males between VBT_60–10_ and VBT_60–30_ (*p* ≤ 0.598 vs. 1.000 and ES ≥ 0.58 vs. ≤ 0.39) and for females between VBT_80–10_ and VBT_80–30_ (*p* ≤ 0.394 vs. 1.000 and ES ≥ 0.75 vs. ≤ 0.13) (Table 1).

**TABLE 1:** Comparison of performance variables between different velocity‐based resistance training (VBT) protocols and sex (11 males and 10 females).

Variable	Sex	Protocol				ANOVA		
		VBT_60‐10_	VBT_60‐30_	VBT_80‐10_	VBT_80‐30_	Protocol	Sex	Interaction
Load (kg)	Males	82.8 ± 17.7	87.5 ± 18.6	113.1 ± 25.4	112.0 ± 24.5	**F**_**(3,57)**_ **= 81.61;**	**F**_**(1,19)**_ **= 27.93;**	**F**_**(3,57)**_ **= 8.41;**
	Females	52.8 ± 8.9	54.4 ± 7.6	66.0 ± 12.7	70.3 ± 10.7	***p* < 0.001**	***p* < 0.001**	***p* < 0.001**
MV_fastest_ (m⋅s^−1^)	Males	0.76 ± 0.07	0.73 ± 0.07	0.54 ± 0.06	0.55 ± 0.07	**F**_**(3,57)**_ **= 73.81;**	F_(1,19)_ = 2.06;	**F**_**(3,57)**_ **= 7.23;**
	Females	0.68 ± 0.04	0.67 ± 0.05	0.58 ± 0.07	0.53 ± 0.05	***p* < 0.001**	*p* = 0.168	***p* < 0.001**
Total number of repetitions	Males	29.4 ± 8.6	51.3 ± 14.4	16.3 ± 3.8	26.1 ± 9.5	**F**_**(3,57)**_ **= 54.26;**	F_(1,19)_ = 0.020;	F_(3,57)_ = 0.13;
	Females	28.9 ± 14.0	53.8 ± 15.1	16.8 ± 7.7	25.2 ± 8.4	***p* < 0.001**	*p* = 0.890	*p* = 0.940

*Note:* Significant differences are emphasized in bold.

Abbreviations: ANOVA, analysis of variance; F, Snedecor's F; MV_fastest_, the fastest mean velocity of the first set; *P*, *p*‐value; VBT_60–10_, VBT protocol with 60% of one‐repetition maximum (1RM) and a velocity loss in the set of 10%; VBT_60–30_, VBT protocol with 60% 1RM and a velocity loss in the set of 30%; VBT_80–10_, VBT protocol with 80% 1RM and a velocity loss in the set of 10%; and VBT_80–30_, VBT protocol with 80% 1RM and a velocity loss in the set of 30%.

### 4.2 Between‐Day Reliability of the L–V Relationship Variables

At the beginning of each session, no differences were reported for *L*_0_ (F_(3,60)_ = 1.31 and *p* = 0.277), *v*_0_ (F_(3,60)_ = 1.35 and *p* = 0.268), and *A*_*line*_ (F_(3,60)_ = 2.46 and *p* = 0.246) between VBT protocols. Additionally, the three L–V relationship variables presented an acceptable between‐day reliability (CV ≤ 5.61% and ICC ≥ 0.83), except for *v*_0_ between VBT_60–30_ and VBT_80–10_ (CV = 5.31 and ICC = 0.78) (Table 2).

**TABLE 2:** Between‐day reliability of the load–velocity (L–V) relationship variables obtained before velocity‐based resistance training (VBT) protocols.

L–V relationship variable	Session	ICC (95% CI)	CV (95% CI) (%)	SEM	SDD
*L*_0_ (kg⋅kg^−1^)	VBT_60–10_ versus VBT_60–30_	0.93 (0.84, 0.97)	5.23 (4.00, 7.55)	0.11	0.30
	VBT_60–10_ versus VBT_80–10_	0.93 (0.84, 0.97)	5.61 (4.29, 8.10)	0.11	0.30
	VBT_60–10_ versus VBT_80–30_	0.95 (0.89, 0.98)	4.44 (3.40, 6.41)	0.09	0.25
	VBT_60–30_ versus VBT_80–10_	0.94 (0.87, 0.98)	4.97 (3.80, 7.17)	0.10	0.27
	VBT_60–30_ versus VBT_80–30_	0.97 (0.92, 0.99)	3.60 (2.75, 5.20)	0.07	0.19
	VBT_80–10_ versus VBT_80–30_	0.96 (0.90, 0.98)	4.37 (3.34, 6.31)	0.09	0.25
*v*_0_ (m⋅s^−1^)	VBT_60–10_ versus VBT_60–30_	0.90 (0.78, 0.96)	4.12 (3.15, 5.95)	0.07	0.19
	VBT_60–10_ versus VBT_80–10_	0.83 (0.63, 0.93)	4.98 (3.81, 7.19)	0.08	0.22
	VBT_60–10_ versus VBT_80–30_	0.92 (0.82, 0.97)	3.67 (2.81, 5.30)	0.06	0.16
	VBT_60–30_ versus VBT_80–10_	**0.78 (0.54, 0.91)**	5.31 (4.06, 7.67)	0.08	0.22
	VBT_60–30_ versus VBT_80–30_	0.90 (0.78, 0.96)	3.92 (3.00, 5.66)	0.06	0.16
	VBT_80–10_ versus VBT_80–30_	0.86 (0.69, 0.94)	4.32 (3.31, 6.24)	0.07	0.19
*A*_*line*_ (kg⋅kg^−1^⋅m⋅s^−1^)	VBT_60–10_ versus VBT_60–30_	0.98 (0.94, 0.99)	4.91 (3.76, 7.10)	0.08	0.22
	VBT_60–10_ versus VBT_80–10_	0.98 (0.96, 0.99)	4.37 (3.35, 6.32)	0.07	0.19
	VBT_60–10_ versus VBT_80–30_	0.98 (0.95, 0.99)	4.82 (3.68, 6.96)	0.02	0.05
	VBT_60–30_ versus VBT_80–10_	0.98 (0.95, 0.99)	4.24 (3.24, 6.12)	0.07	0.19
	VBT_60–30_ versus VBT_80–30_	0.98 (0.96, 0.99)	3.91 (2.99, 5.64)	0.06	0.16
	VBT_80–10_ versus VBT_80–30_	0.99 (0.98, 1.00)	3.03 (2.32, 4.38)	0.05	0.14

*Note:* Bold numbers indicate an unacceptable reliability (CV > 10% and ICC < 0.80).

Abbreviations: 95% CI, 95% confidence interval; *A*_*line*_, area under the L–V relationship line; CV, coefficient of variation; ICC, intraclass correlation coefficient; *L*_*0*_, load‐axis intercept; SEM, standard error of measurement; SDD, smallest detectable difference; *v*_0_, velocity‐axis intercept; VBT_60–10_, VBT protocol with 60% of one‐repetition maximum (1RM) and a velocity loss in the set of 10%; VBT_60–30_, VBT protocol with 60% 1RM and a velocity loss in the set of 30%; VBT_80–10_, VBT protocol with 80% 1RM and a velocity loss in the set of 10%; and VBT_80–30_, VBT protocol with 80% 1RM and a velocity loss in the set of 30%.

### 4.3 Changes in the L–V Relationship Variables

**FIGURE 1:**
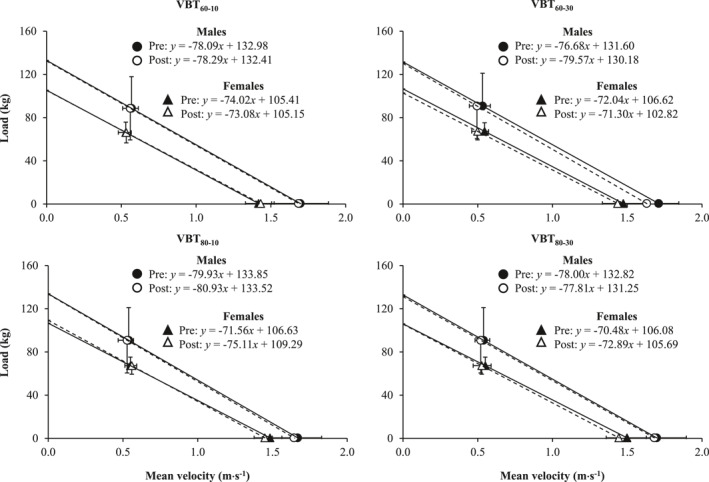
Changes in the load–velocity profile after different velocity‐based resistance training (VBT) protocols separately for males (*n* = 11; circles) and females (*n* = 10; triangles). Black and white symbols represent the load–velocity profiles obtained before and after each VBT protocol, respectively. The regression equations are shown in each panel. VBT_60–10_, VBT protocol with 60% of one‐repetition maximum (1RM) and a velocity loss in the set of 10%; VBT_60–30_, VBT protocol with 60% 1RM and a velocity loss in the set of 30%; VBT_80–10_, VBT protocol with 80% 1RM and a velocity loss in the set of 10%; and VBT_80–30_, VBT protocol with 80% 1RM and a velocity loss in the set of 30%.

A main effect of time was observed for *v*_0_ (F_(1,19)_ = 6.74 and *p* = 0.018) and *A*_*line*_ (F_(1,19)_ = 30.20 and *p* < 0.001), with both variables reduced after the VBT protocols (ES = −0.39 and −0.48, respectively). A main effect for sex was also noted for *v*_0_ (F_(1,19)_ = 14.50 and *p* = 0.001), with greater magnitude for males than for females (ES = 1.63) (Figure 1). A protocol × time interaction was noted for *v*_0_ (F_(1,19)_ = 2.94 and *p* = 0.041) and *A*_*line*_ (F_(1,19)_ = 5.10 and *p* = 0.003). Both *v*_0_ and *A*_*line*_ were reduced after VBT_60–30_ (*p* = 0.013 and < 0.001 and ES = −0.59 and −1.10, respectively) and VBT_80–30_ (*p* = 0.044 and 0.017 and ES = −0.47 and −0.57, respectively) but not after VBT_60–10_ (*p* = 0.931 and 0.066 and ES = 0.02 and −0.10, respectively) and VBT_80–10_ (*p* = 0.108 and 0.425 and ES = −0.37 and −0.18, respectively). Statistical significance was not reached for the remaining main effects and interactions (Table 3).

**TABLE 3:** Comparison of the load–velocity (L–V) relationship variables obtained before and after velocity‐based resistance training (VBT) protocols for each sex (11 males and 10 females).

L‐V relationship variable	Protocol (P)	Sex (S)	Time (T)		ANOVA
			Pre	Post	
*L*_0_ (kg⋅kg^−1^)	VBT_60–10_	Males	2.10 ± 0.47	2.09 ± 0.45	P: F_(3,57)_ = 0.48; *p* = 0.695 T: F_(1,19)_ = 1.05; *p* = 0.319 S: F_(1,19)_ = 0.91; *p* = 0.353 P × T: F_(3,57)_ = 2.66; *p* = 0.057 P × S: F_(3,57)_ = 0.57; *p* = 0.638 T × S: F_(1,19)_ = 0.09; *p* = 0.770 P × T × S: F_(3,19)_ = 1.04; *p* = 0.381
		Females	1.93 ± 0.29	1.92 ± 0.28	
	VBT_60–30_	Males	2.08 ± 0.46	2.05 ± 0.41	
		Females	1.96 ± 0.27	1.89 ± 0.26	
	VBT_80–10_	Males	2.08 ± 0.49	2.08 ± 0.47	
		Females	1.85 ± 0.30	1.91 ± 0.35	
	VBT_80–10_	Males	2.07 ± 0.45	2.04 ± 0.47	
		Females	1.93 ± 0.34	1.92 ± 0.34	
*v*_0_ (m⋅s^−1^)	VBT_60–10_	Males	1.70 ± 0.19	1.69 ± 0.18	P: F_(3,57)_ = 0.69; *p* = 0.562 **T: F**_**(1,19)**_ **= 6.74; *p* = 0.018** **S:** **F**_**(1,19)**_ **= 14.50; *p* = 0.001** **P × T:** **F**_**(3,57)**_ **= 2.94; *p* = 0.041** P × S: F_(3,57)_ = 1.80; *p* = 0.158 T × S: F_(1,19)_ = 0.04; *p* = 0.846 P × T × S: F_(3,19)_ = 1.76; *p* = 0.165
		Females	1.42 ± 0.11	1.44 ± 0.10	
	VBT_60–30_	Males	1.72 ± 0.13	1.63 ± 0.18	
		Females	1.48 ± 0.17	1.44 ± 0.10	
	VBT_80–10_	Males	1.67 ± 0.16	1.65 ± 0.14	
		Females	1.49 ± 0.08	1.46 ± 0.07	
	VBT_80–10_	Males	1.70 ± 0.20	1.69 ± 0.19	
		Females	1.51 ± 0.13	1.45 ± 0.08	
*A*_*line*_ (kg⋅kg^−1^⋅m⋅s^−1^)	VBT_60–10_	Males	1.82 ± 0.61	1.79 ± 0.57	P: F_(3,57)_ = 0.75; *p* = 0.530 **T: F**_**(1,19)**_ **= 30.20; *p* < 0.001** S: F_(1,19)_ = 3.65; *p* = 0.071 **P × T:** **F**_**(3,57)**_ **= 5.10; *p* = 0.003** P × S: F_(3,57)_ = 1.39; *p* = 0.256 T × S: F_(1,19)_ = 1.33; *p* = 0.263 P × T × S: F_(3,19)_ = 0.59; *p* = 0.625
		Females	1.38 ± 0.25	1.39 ± 0.26	
	VBT_60–30_	Males	1.81 ± 0.53	1.70 ± 0.53	
		Females	1.45 ± 0.25	1.36 ± 0.23	
	VBT_80–10_	Males	1.77 ± 0.57	1.73 ± 0.51	
		Females	1.39 ± 0.25	1.39 ± 0.29	
	VBT_80–10_	Males	1.79 ± 0.59	1.76 ± 0.60	
		Females	1.45 ± 0.28	1.39 ± 0.27	

*Note*: Significant differences are emphasized in bold.

Abbreviations: ANOVA, analysis of variance; *A*_*line*_, area under the L–V relationship line; CV, coefficient of variation; F, Snedecor's F; *L*_*0*_, load‐axis intercept; *v*_0_, velocity‐axis intercept; *P*, *p*‐value; VBT_60–10_, VBT protocol with 60% of one‐repetition maximum (1RM) and a velocity loss in the set of 10%; VBT_60–30_, VBT protocol with 60% 1RM and a velocity loss in the set of 30%; VBT_80–10_, VBT protocol with 80% 1RM and a velocity loss in the set of 10%; and VBT_80–30_, VBT protocol with 80% 1RM and a velocity loss in the set of 30%.

**FIGURE 2:**
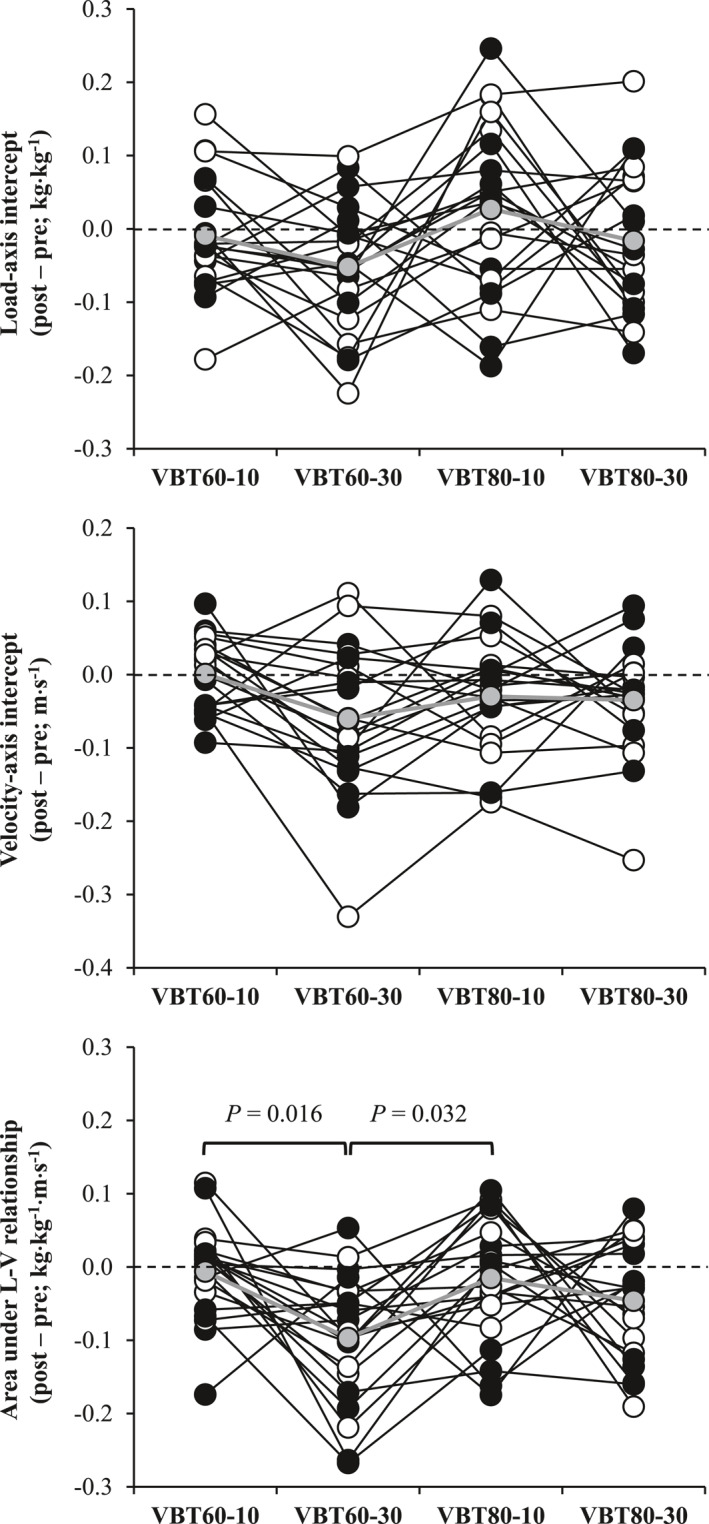
Individual changes in the load‐axis intercept (upper panel), velocity‐axis intercept (middle panel), and area under the load–velocity (L–V) relationship area (lower panel) following different velocity‐based resistance training (VBT) protocols in males (*n* = 11; black circles) and females (*n* = 10; white circles). Gray circles and lines represent the magnitude of the changes averaged across the subjects. VBT_60–10_, VBT protocol with 60% of one‐repetition maximum (1RM) and a velocity loss in the set of 10%; VBT_60–30_, VBT protocol with 60% 1RM and a velocity loss in the set of 30%; VBT_80–10_, VBT protocol with 80% 1RM and a velocity loss in the set of 10%; and VBT_80–30_, VBT protocol with 80% 1RM and a velocity loss in the set of 30%. *P*, *p*‐value (analysis of variance with Bonferroni correction).

The ANOVA carried out on the post–pre differences revealed a main effect for *v*_0_ (F_(3,60)_ = 2.93 and *p* = 0.041) and *A*_*line*_ (F_(3,60)_ = 5.11 and *p* = 0.003) but not for *L*_0_ (F_(3,60)_ = 2.61 and *p* = 0.060). Although *v*_0_ tended to be reduced to a greater extent for VBT_60–30_ than for VBT_60–10_ (*p* = 0.097 and ES = −0.57), the reduction in *A*_*line*_ was greater for the VBT_60–30_ than for VBT_60–10_ (*p* = 0.016 and ES = −0.75) and VBT_80–10_ (*p* = 0.032 and ES = −0.67) (Figure 2). Post–pre differences were not associated between VBT protocols for the *L*_0_ (*r* ≤ 0.204 and *p* ≥ 0.376), *v*_0_ (*r* ≤ −0.201 and *p* ≥ 0.383), and *A*_*line*_ (*r* ≤ −0.327 and *p* ≥ 0.148).

## 5 Discussion

This study was designed to explore the feasibility of L–V relationship variables obtained during the countermovement jump exercise to monitor acute changes in the lower‐body maximal mechanical capacities, following different back squat VBT protocols (VBT_60–10_ vs. VBT_60–30_ vs. VBT_80–10_ vs. VBT_80–30_) in male and female recreational runners. Main results revealed that (i) the three L–V relationship variables (*L*_0_, *v*_0_, and *A*_*line*_) presented an acceptable reliability in four successive sessions, (ii) both *v*_0_ and *A*_*line*_ were reduced after the VBT_60–30_ and VBT_80–30_ protocols, being the post–pre differences of *A*_*line*_ greater for VBT_60–30_ than for VBT_60–10_ and VBT_80–10_, (iii) the changes in the L–V relationship variables were comparable between sexes, and (iv) the post–pre differences for each L–V relationship variable was not associated between the different VBT protocols.

An open scientific debate concerns the reliability of F–V relationship outcomes to evaluate the maximal neuromuscular capacities obtained during multijoint maximum performance tasks (Samozino et al. 2022). The reliability of F–V relationship parameters depends on factors such as participants’ characteristics (e.g., task experience) and the testing procedures (Fessl et al. 2022; Lindberg et al. 2021; Pérez‐Castilla et al. 2018). Particularly, the distance of the variable of interest to the closest experimental point is a key characteristic to obtain the F–V relationship parameters with reliability and precision (García‐Ramos 2023). As force outputs are incorporated into the modeling, in resistance training exercises performed against gravity (e.g., countermovement jump), the reliability of F–V relationship parameters is compromised by the large extrapolation from the experimental point to *v*_0_ (Pérez‐Castilla et al. 2021). As a methodological alternative, the L–V relationship may be used in replace of the F–V relationship (Iglesias‐Soler et al. 2021; Pérez‐Castilla et al. 2021; Pérez‐Castilla et al. 2022). Indeed, current results corroborate the high reliability of the three variables derived from the L–V relationship to evaluate the maximal mechanical capacities of the lower‐body muscles on successive occasions, in line with previous studies (Pérez‐Castilla et al. 2022; Pérez‐Castilla, Ramirez‐Campillo, et al. 2023), and supporting our first hypothesis. Moreover, the L–V relationship was obtained from the two‐point method applied under field conditions. This finding confirms the high between‐session reliability of the two‐point method observed by Miras‐Moreno et al. (2023) during the prone bench pull exercise performed in a Smith machine. In summary, current results provide additional evidence of the two‐point method as a simple, fast, and reliable procedure for determining the lower‐body maximal mechanical capacities through the L–V relationship. However, practitioners and coaches should keep in mind the larger SDD values when interpreting meaningful changes in performance through the L–V relationship in recreational runners.

The L–V relationship variables can be used to assess mechanical fatigue across the velocity spectrum following physical exertion (García‐Ramos et al. 2018; Morenas‐Aguilar et al. 2023; Quidel‐Catrilelbún et al. 2024). This practical application is based on studies showing high sensitivity of the F–V relationship to selectively detect acute changes in the function of the upper body (García‐Ramos et al. 2018) and lower body (Li et al. 2024) muscles following different resistance training protocols. Applying this novel approach, our second hypothesis was rejected since *A*_*line*_ reduction was more predominant for high‐repetition VBT protocols (VBT_60–30_ and VBT_80–30_) with selective deterioration of *v*_0_ instead of *L*_0_. Furthermore, the post–pre differences of *A*_*line*_ were greater for VBT_60–30_ than for VBT_60–10_ and VBT_80–10_. This finding is contrary to the results reported by Quidel‐Catrilelbun et al. (2023). The authors (Quidel‐Catrilelbún et al. 2024) determined the L–V relationship during the prone bench pull exercise to quantify fatigue induced using the same VBT protocols (VBT_60–10_, VBT_60–30_, VBT_80–10_, and VBT_80–30_) in competitive rowers. Rowing is characterized by a force‐orientated F–V profile (Giroux et al. 2017; Pérez‐Castilla, Quidel‐Catrilelbún, et al. 2023), and running is characterized by a velocity‐orientated F–V profile (Sabater Pastor et al. 2023), suggesting sport‐dependent maximal mechanical capabilities. Additionally, the impairment of maximal neuromuscular capacities may depend on the exercise selection (i.e., prone bench pull vs. countermovement jump) (de‐Oliveira et al. 2023). Moreover, the L–V relationship in the study by Quidel‐Catrilelbun et al. (2023) was determined after each VBT protocol and a 2000 m rowing ergometer time trial. Therefore, although the L–V relationship appears to be sensitive to detect acute changes in the specific maximal mechanical capacities following different VBT protocols in different sporting disciplines (e.g., rowing and running), future studies need to confirm the suitability of this novel approach in different sports.

Females showed greater muscle fatigability than men (Rissanen et al. 2022; Walker et al. 2022), probably due to the lower muscle mass and greater whole muscle area of type I fibers (Nuzzo 2023). However, rejecting our third hypothesis, the changes in the L–V profile were comparable between sexes. This result agrees with the same number of total repetitions reported for males and females training with the same velocity loss. Since resistance‐untrained subjects appear to report greater muscle damage and attenuation than resistance‐trained subjects (Skurvydas et al. 2011), it seems logical to speculate that the greater muscular strength reported in the present study for males (back squat 1RM relative to body mass = 1.8 vs. 1.5) has been offset by the greater fatigability attributed in the literature for females. Future studies should explore the acute and chronic impact of different VBT protocols on males and females with similar training status and history to shed more light on this question.

This study offers new insights into the practical utility of the L–V relationship. However, caution should be exercised when interpreting current findings, due to the high intra‐individual variability observed when the post–pre differences of each L–V relationship variable was associated between the different VBT protocols (see Figure 2). Li et al. (2024) reported the individual changes in the vertical F–V relationship following a heavy‐load traditional (five repetitions at 80% 1RM) and light‐load ballistic (five repetitions at 30% 1RM) protocol. These authors (Li et al. 2024) reported that subjects increased or decreased maximal force capacity (*F*_*0*_) and *v*_0_ following the light‐load ballistic and heavy‐load traditional protocols, respectively. This high interindividual variability was observed even though Li et al. (2024) used a more controlled testing procedure to determine the F–V relationship (squat jumps under four loading conditions) than that used in the present study to determine the L–V relationship (countermovement jumps under two loading conditions). Therefore, unlike previous studies (García‐Ramos et al. 2018; Li et al. 2024), we cannot confirm the feasibility of the F–V profile to detect acute effects of different fatigue protocols because the individual response seems to be highly variable, that is, the same subject may experience fatigue or potentiation after VBT protocols with similar characteristics. Future studies should examine the relationship between changes in the L–V and F–V profile and certain markers of metabolic stress (e.g., lactate and ammonia) to yield a more comprehensive understanding of the underlying mechanisms noted in this study. Additionally, future research should investigate the feasibility of the L–V and F–V profiles in real‐world contexts, where a variety of lower‐body and upper‐body exercises are incorporated into resistance training sessions. From a practical perspective, alternative testing procedures are recommended to assess fatigue‐induced changes in runners. For example, the isometric force (maximal voluntary contractions of knee extensors and plantar flexors) deteriorates 2–3 times more than the F–V relationship parameters (maximal 7‐s sprints on a cycle ergometer) after trail running races ranging from 40 to 170 km (Koral et al. 2021).

## 6 Conclusions

Coaches and strength and conditioning professionals should handle the residual fatigue induced by resistance training sessions to attenuate the RT‐SEP phenomenon in runners. Our data corroborate the high between‐session reliability of the L–V relationship variables obtained during the countermovement jump exercise under field conditions to evaluate the lower‐body maximal mechanical variables across successive sessions. However, practitioners and coaches should take into account the SDD reported in this study when interpreting meaningful changes in the L–V profiles of recreational runners. In general, the L–V relationship also seems to be sufficiently sensitive to detect acute changes in the specific maximal mechanical capacities following different VBT protocols. Specifically, *v*_0_ and *A*_*line*_ were significantly reduced after high‐repetition VBT protocols, being those changes comparable between males and females. However, caution should be taken when interpreting current findings and analyzing each change on a case‐by‐case basis due to the high variability observed in individual responses.

## Conflicts of Interest

The authors declare no conflicts of interest.

## References

[ejsc12309-bib-0001] Baena‐Raya, A. , D. M. Díez‐Fernández , A. García‐de‐Alcaraz , A. Soriano‐Maldonado , A. Pérez‐Castilla , and M. A. Rodríguez‐Pérez . 2024. “Assessing the Maximal Mechanical Capacities through the Load‐Velocity Relationship in Elite versus Junior Male Volleyball Players.” Sport Health: A Multidisciplinary Approach 6, no. 5: 829–836. 10.1177/19417381231208706. PMC11346240 37950435

[ejsc12309-bib-0002] Beattie, K. , B. P. Carson , M. Lyons , A. Rossiter , and I. C. Kenny . 2017. “The Effect of Strength Training on Performance Indicators in Distance Runners.” Journal of Strength & Conditioning Research 31, no. 1: 9–23. 10.1519/JSC.0000000000001464. 27135468

[ejsc12309-bib-0003] Besson, T. , R. Macchi , J. Rossi , et al. 2022. “Sex Differences in Endurance Running.” Sports Medicine 52, no. 6: 1235–1257. 10.1007/s40279-022-01651-w. 35122632

[ejsc12309-bib-0004] Blagrove, R. C. , G. Howatson , and P. R. Hayes . 2018. “Effects of Strength Training on the Physiological Determinants of Middle‐ and Long‐Distance Running Performance: A Systematic Review.” Sports Medicine 48, no. 5: 1117–1149. 10.1007/s40279-017-0835-7. 29249083 PMC5889786

[ejsc12309-bib-0005] de Carvalho e Silva, G. I. , L. H. A. Brandão , D. dos Santos Silva , et al. 2022. “Acute Neuromuscular, Physiological and Performance Responses After Strength Training in Runners: A Systematic Review and Meta‐Analysis.” Sports Medicine ‐ Open 8, no. 1: 105. 10.1186/s40798-022-00497-w. 35976540 PMC9385928

[ejsc12309-bib-0006] de‐Oliveira, L. A. , J. C. Aragão‐Santos , J. R. Heredia‐Elvar , and M. E. Da Silva‐Grigoletto . 2023. “Movement Velocity as an Indicator of Mechanical Fatigue and Resistance Exercise Intensity in Cross Modalities.” Research Quarterly for Exercise & Sport 94, no. 4: 1028–1034. 10.1080/02701367.2022.2101603. 36006785

[ejsc12309-bib-0007] Doma, K. , G. B. Deakin , and D. J. Bentley . 2017. “Implications of Impaired Endurance Performance Following Single Bouts of Resistance Training: An Alternate Concurrent Training Perspective.” Sports Medicine 47, no. 11: 2187–2200. 10.1007/s40279-017-0758-3. 28702901

[ejsc12309-bib-0008] Doma, K. , G. B. Deakin , M. Schumann , and D. J. Bentley . 2019. “Training Considerations for Optimising Endurance Development: An Alternate Concurrent Training Perspective.” Sports Medicine 49, no. 5: 669–682. 10.1007/s40279-019-01072-2. 30847824

[ejsc12309-bib-0009] Fessl, I. , H.‐P. Wiesinger , and J. Kröll . 2022. “Power–Force–Velocity Profiling as a Function of Used Loads and Task Experience.” International Journal of Sports Physiology and Performance 17, no. 5: 694–700. 10.1123/ijspp.2021-0325. 35158325

[ejsc12309-bib-0010] García‐Ramos, A. 2023. “The 2‐Point Method: Theoretical Basis, Methodological Considerations, Experimental Support, and its Application Under Field Conditions.” International Journal of Sports Physiology and Performance 18, no. 10: 1092–1100. 10.1123/ijspp.2023-0127. 37541677

[ejsc12309-bib-0011] García‐Ramos, A. , A. Torrejón , B. Feriche , et al. 2018. “Selective Effects of Different Fatigue Protocols on the Function of Upper Body Muscles Assessed through the Force–Velocity Relationship.” European Journal of Applied Physiology 118, no. 2: 439–447. 10.1007/s00421-017-3786-7. 29242994

[ejsc12309-bib-0012] Giroux, C. , H. Maciejewski , A. Ben‐Abdessamie , et al. 2017. “Relationship Between Force‐Velocity Profiles and 1,500‐m Ergometer Performance in Young Rowers.” International Journal of Sports Medicine 38, no. 13: 992–1000. 10.1055/s-0043-117608. 28965345

[ejsc12309-bib-0013] Hopkins, W. G. 2015. “Spreadsheets for Analysis of Validity and Reliability.” Sportscience 19: 36–42: doi: sportsci.org/2017/wghxls.htm.

[ejsc12309-bib-0014] Hopkins, W. G. , S. W. Marshall , A. M. Batterham , and J. Hanin . 2009. “Progressive Statistics for Studies in Sports Medicine and Exercise Science.” Medicine & Science in Sports & Exercise 41, no. 1: 3–13. 10.1249/MSS.0b013e31818cb278. 19092709

[ejsc12309-bib-0015] Iglesias‐Soler, E. , J. Rial‐Vázquez , D. Boullosa , et al. 2021. “Load‐velocity Profiles Change After Training Programs With Different Set Configurations.” International Journal of Sports Medicine 42, no. 09: 794–802. 10.1055/a-1323-3456. 33352601

[ejsc12309-bib-0016] Jaric, S. 2016. “Two‐Load Method for Distinguishing Between Muscle Force, Velocity, and Power‐Producing Capacities.” Sports Medicine 46, no. 11: 1585–1589. 10.1007/s40279-016-0531-z. 27075326 PMC5056118

[ejsc12309-bib-0017] Jung, A. P. 2003. “The Impact of Resistance Training on Distance Running Performance.” Sports Medicine 33, no. 7: 539–552. 10.2165/00007256-200333070-00005. 12762828

[ejsc12309-bib-0018] Koral, J. , M. Fanget , L. Imbert , et al. 2021. “Fatigue Measured in Dynamic versus Isometric Modes After Trail Running Races of Various Distances.” International Journal of Sports Physiology and Performance 17, no. 1: 67–77. 10.1123/ijspp.2020-0940. 34359049

[ejsc12309-bib-0019] Li, Z. , P. Zhi , Z. Yuan , A. García‐Ramos , and M. King . 2024. “Feasibility of Vertical Force–Velocity Profiles to Monitor Changes in Muscle Function Following Different Fatigue Protocols.” European Journal of Applied Physiology 124, no. 1: 365–374. 10.1007/s00421-023-05283-4. 37535143

[ejsc12309-bib-0020] Lindberg, K. , P. Solberg , T. Bjørnsen , et al. 2021. “Force‐velocity Profiling in Athletes: Reliability and Agreement Across Methods.” PLoS One 16, no. 2: e0245791. 10.1371/journal.pone.0245791. 33524058 PMC7850492

[ejsc12309-bib-0021] Miras‐Moreno, S. , A. García‐Ramos , I. Jukic , and A. Pérez‐Castilla . 2023. “Two‐point Method Applied in Field Conditions: A Feasible Approach to Assess the Load‐Velocity Relationship Variables During the Bench Pull Exercise.” Journal of Strength & Conditioning Research 37, no. 7: 1367–1474. 10.1519/JSC.0000000000004405. 36728020

[ejsc12309-bib-0022] Morenas‐Aguilar, M. D. , S. A. Ruiz‐Alias , A. M. Blanco , C. Lago‐Fuentes , F. García‐Pinillos , and A. Pérez‐Castilla . 2023. “Does the Menstrual Cycle Impact the Maximal Neuromuscular Capacities of Women? An Analysis Before and After a Graded Treadmill Test to Exhaustion.” Journal of Strength & Conditioning Research 37, no. 11: 2185–2191. 10.1519/JSC.0000000000004542. 37883397

[ejsc12309-bib-0023] Nuzzo, J. L. 2023. “Narrative Review of Sex Differences in Muscle Strength, Endurance, Activation, Size, Fiber Type, and Strength Training Participation Rates, Preferences, Motivations, Injuries, and Neuromuscular Adaptations.” Journal of Strength & Conditioning Research 37, no. 2: 494–536. 10.1519/JSC.0000000000004329. 36696264

[ejsc12309-bib-0024] Pérez‐Castilla, A. , A. García‐Ramos , P. Padial , A. J. Morales‐Artacho , and B. Feriche . 2020. “Load‐Velocity Relationship in Variations of the Half‐Squat Exercise: Influence of Execution Technique.” Journal of Strength & Conditioning Research 34, no. 4: 1024–1031. 10.1519/JSC.0000000000002072. 28885389

[ejsc12309-bib-0025] Pérez‐Castilla, A. , S. Jaric , B. Feriche , P. Padial , and A. García‐Ramos . 2018. “Evaluation of Muscle Mechanical Capacities through the Two‐Load Method: Optimization of the Load Selection.” Journal of Strength & Conditioning Research 32, no. 5: 1245–1253. 10.1519/JSC.0000000000001969. 28475551

[ejsc12309-bib-0026] Pérez‐Castilla, A. , I. Jukic , and A. García‐Ramos . 2021. “Validation of a Novel Method to Assess Maximal Neuromuscular Capacities through the Load‐Velocity Relationship.” Journal of Biomechanics 127: 110684. 10.1016/j.jbiomech.2021.110684. 34416531

[ejsc12309-bib-0027] Pérez‐Castilla, A. , I. Jukic , D. Janicijevic , Z. Akyildiz , D. Senturk , and A. García‐Ramos . 2022. “Load‐Velocity Relationship Variables to Assess the Maximal Neuromuscular Capacities During the Back‐Squat Exercise.” Sport Health: A Multidisciplinary Approach 14, no. 6: 885–893. 10.1177/19417381211064603. PMC9631046 35114871

[ejsc12309-bib-0028] Pérez‐Castilla, A. , A. Piepoli , G. Delgado‐García , G. Garrido‐Blanca , and A. García‐Ramos . 2019. “Reliability and Concurrent Validity of Seven Commercially Available Devices for the Assessment of Movement Velocity at Different Intensities During the Bench Press.” Journal of Strength & Conditioning Research 33, no. 5: 1258–1265. 10.1519/JSC.0000000000003118. 31034462

[ejsc12309-bib-0029] Pérez‐Castilla, A. , M. E. L. Quidel‐Catrilelbún , M. A. Rodríguez‐Pérez , and A. García‐Ramos . 2023. “Association of the Load‐Velocity Relationship Variables with 2000‐m Rowing Ergometer Performance.” European Journal of Sport Science 23, no. 5: 1–10. 10.1080/17461391.2022.2054364. 35290158

[ejsc12309-bib-0030] Pérez‐Castilla, A. , R. Ramirez‐Campillo , J. F. T. Fernandes , and A. García‐Ramos . 2023. “Feasibility of the 2‐point Method to Determine the Load−velocity Relationship Variables During the Countermovement Jump Exercise.” Journal of Sport and Health Science 12, no. 4: 544–552. 10.1016/j.jshs.2021.11.003. 34852294 PMC10362485

[ejsc12309-bib-0031] Quidel‐Catrilelbún, M. E. L. , S. A. Ruiz‐Alias , F. García‐Pinillos , R. Ramirez‐Campillo , and A. Pérez‐Castilla . 2024. “Acute Effect of Different Velocity‐Based Training Protocols on 2000‐meter Rowing Ergometer Performance.” Journal of Strength & Conditioning Research 38, no. 1: e8–e15. 10.1519/JSC.0000000000004595. 38085632

[ejsc12309-bib-0032] Rissanen, J. , S. Walker , F. Pareja‐Blanco , and K. Häkkinen . 2022. “Velocity‐based Resistance Training: Do Women Need Greater Velocity Loss to Maximize Adaptations?” European Journal of Applied Physiology 122, no. 5: 1269–1280. 10.1007/s00421-022-04925-3. 35258681 PMC9012837

[ejsc12309-bib-0033] Sabater Pastor, F. , T. Besson , M. Berthet , et al. 2023. “Elite Road vs. Trail Runners: Comparing Economy, Biomechanics, Strength, and Power.” Journal of Strength & Conditioning Research 37, no. 1: 181–186. 10.1519/JSC.0000000000004226. 36515604

[ejsc12309-bib-0034] Samozino, P. , J. R. Rivière , P. Jimenez‐Reyes , M. R. Cross , and J.‐B. Morin . 2022. “Is the Concept, Method, or Measurement to Blame for Testing Error? An Illustration Using the Force‐Velocity‐Power Profile.” International Journal of Sports Physiology and Performance 17, no. 12: 1760–1768. 10.1123/ijspp.2021-0535. 36368326

[ejsc12309-bib-0035] Skurvydas, A. , M. Brazaitis , T. Venckūnas , S. Kamandulis , A. Stanislovaitis , and A. Zuoza . 2011. “The Effect of Sports Specialization on Musculus Quadriceps Function After Exercise‐Induced Muscle Damage.” Applied Physiology Nutrition and Metabolism 36, no. 6: 873–880. 10.1139/h11-112. 22050132

[ejsc12309-bib-0036] Walker, S. , K. Häkkinen , R. Virtanen , S. Mane , B. Bachero‐Mena , and F. Pareja‐Blanco . 2022. “Acute Neuromuscular and Hormonal Responses to 20 versus 40% Velocity Loss in Males and Females Before and After 8 Weeks of Velocity‐loss Resistance Training.” Experimental Physiology 107, no. 9: 1046–1060. 10.1113/EP090371. 35930559 PMC9542169

